# New Appliances and Things Medical

**Published:** 1899-02-18

**Authors:** 


### NEW APPLIANCES AND THINGS MEDICAL.

[We shall be glad to receive, at our Office 28 & 29, Southampton Street, Strand, London, W.C., from the manufacturers, specimens of all new preparations and appliances which may be brought out from time to time.]

### IMPROVED MEDICAL BATTERY AND ACCESSORIES.

We regret that in our notice of these batteries the address of the makers, Messrs. Niblett and Sutherland, was given as being 31 instead of 61, Chandos Street, Strand.

